# The brightest stars - a tribute to Moritz Kircher

**DOI:** 10.7150/ntno.63531

**Published:** 2022-01-01

**Authors:** Chrysafis Andreou

**Affiliations:** Head of Nanotechnology Imaging and Detection Laboratory, Department of Electrical and Computer Engineering, University of Cyprus, Nicosia, Cyprus

Whenever I think of Moritz Kircher, the shape of the star forms in my mind. Most of the work we did together focused on gold nanostars applied to the imaging and detection of cancer via the effect we call surface enhanced resonance Raman scattering. You see, because of their shape, with the many sharp rays, gold nanostars focus laser light and re-emit it as a bright and distinct signal which can be used to reveal even microscopic tumors. He was so proud of the nanostars his lab produced—among the brightest optical contrast agents used in preclinical imaging.

Moritz was also regarded as somewhat of a star in the field of nanotheranostics and imaging. His career was nothing less than stellar. He graduated with the highest honors from Humboldt University in Berlin. He then completed a string of very successful fellowships in leading institutions in the US, including the Beth Israel Deaconess Medical Center, Stanford University, and the Massachusetts General Hospital. I met him when he was a faculty member of Memorial Sloan Kettering Cancer Center. There, he served many roles and held many responsibilities; I always wondered if he ever had time to sleep. In addition to running his lab, he held a long list of titles and affiliation including Associate Professor at Cornell, an Associate Member at MSKCC, Associate Attending Radiologist in the Body Imaging Service, and Associate Vice Chair for Research in the Department of Radiology, among others. He served as the founding chair of the "Molecular Imaging in Nanotechnology and Theranostics" (MINT) Interest Group of the World Molecular Imaging Society and as the Editor-in-Chief of the journal* Nanotheranostics.* For his final position, Moritz moved to Boston and started as the Chair of the Dana-Farber Cancer Institute Department of Imaging and Radiology and as Chief of the Oncoradiology Division of the Brigham and Women's Radiology Department.

As a researcher, Moritz left a shining mark in the field of nanomedicine. Through his research he developed new nanoparticle agents for medical imaging and therapy. He focused on optical modalities - using light for medical imaging - and became a pioneer in Raman and optoacoustic imaging. Moritz, along with Sam Gambhir, was at the forefront of the clinical translation of these technologies. Moritz also pushed for the development of theranostic agents, combining imaging, diagnosis, and therapy in a single construct. He contributed to these fields with almost two hundred papers and patents. His works received more than 7,000 citations.

Moritz was a bright scientist and an inspiring advisor. Ambition and determination characterized his research, and he instilled these traits in his trainees. Nearly all of us under his guidance moved on to our chosen career paths, propelled by our accomplishments in his lab. His passion for our projects inspired us to work harder and pursue ever more ambitious experiments and goals. In his lab it seemed that every project was successful, even the most difficult in vivo studies. It was as if, in the face of any difficulty, Moritz just refused to accept defeat, and would only settle for a final, brilliant outcome.

It may be that his refusal to accept defeat led to his tragic and untimely demise. Struck by circumstance and adversity, he faced an unfamiliar trial; and for the last time, he refused to suffer defeat. The news of his death left us shocked, with a void in our hearts and many a question in our minds. It is said that the brightest stars burn the fastest. Moritz was among them—a brilliant scientist, blazing a luminous path across the dark sky.

When seeing a shooting star, children often make a wish. Some of us who caught a glimpse of Moritz's light, wish that his trajectory was not quite so short. My own wish is that we continue along the radiant path that he has carved for us and advance his legacy, the translation of nanotheranostic agents into the clinic.

